# Supplementary material for: First-order assessment of flood vulnerability in the Chignecto Isthmus, Atlantic Canada
Source: Camb Prism Coast Futur. 2026 Feb 27;4:e4. doi: 10.1017/cft.2026.10024 (PMC12964069; doi:10.1017/cft.2026.10024)
Supplement: Tackley et al. supplementary material [file S2754720526100249sup001.pdf]

Supplementary material for the article First-Order Assessment of Flood Vulnerability in the Chignecto Isthmus, Atlantic Canada

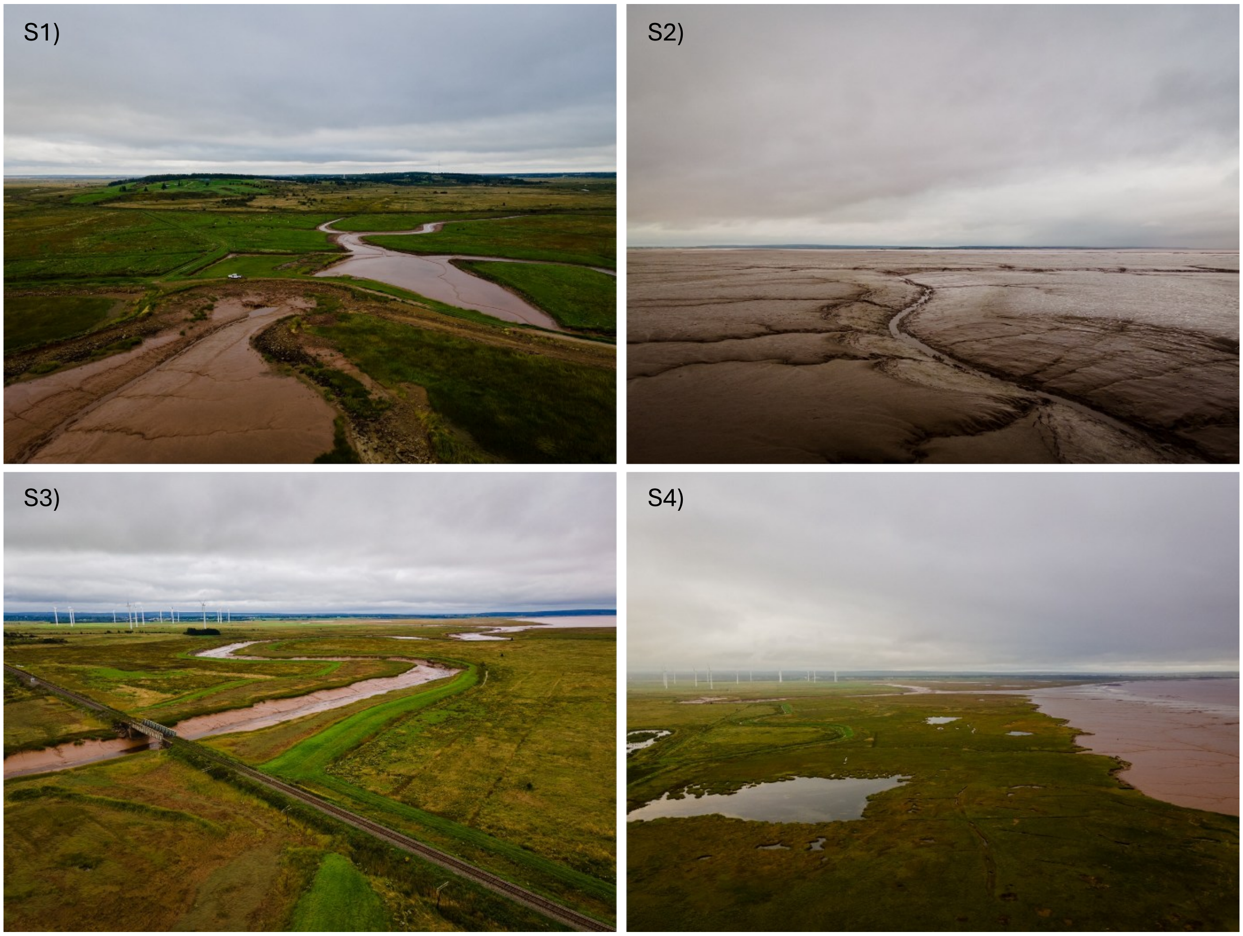

Supplementary Figure: Field photographs from the Chignecto Isthmus illustrating low-lying terrain, infrastructure, and land use. (a) Earthen dike near Aulac protecting agricultural land; the structure is typical of legacy flood infrastructure in the region. (b) Tidal mudflat exposed at low tide, representative of the intertidal environments bordering the isthmus. (c) Rail corridor crossing dikeland, with dike segments in the foreground and wind turbines visible in the background. (d) Coastal agricultural land near the shoreline, with adjacent dikes and distant wind energy infrastructure. These images underscore the region's flat topography, proximity of infrastructure to potential flood zones, and mixed land use across the floodplain.
